# Identification of circulating metabolites linked to the risk of breast cancer: a mendelian randomization study

**DOI:** 10.3389/fphar.2024.1442723

**Published:** 2024-09-11

**Authors:** Xiaosheng Zhu, Huai Huang, Mengjie Zou, Honglin Luo, Tianqi Liu, Shaoliang Zhu, Bin Ye

**Affiliations:** ^1^ Department of Radiation, Jiangbin Hospital of Guangxi Zhuang Autonomous Region, Nanning, China; ^2^ Department of Cardiology, The People’s Hospital of Guangxi Zhuang Autonomous Region, Guangxi Academy of Medical Sciences, Nanning, China; ^3^ Department of Nephrology, The People’s Hospital of Guangxi Zhuang Autonomous Region, Guangxi Academy of Medical Sciences, Nanning, China; ^4^ Institute of Oncology, The People’s Hospital of Guangxi Zhuang Autonomous Region, Guangxi Academy of Medical Sciences, Nanning, China; ^5^ Department of General Surgery, Jiangbin Hospital of Guangxi Zhuang Autonomous Region, Nanning, China; ^6^ Department of Hepatobiliary, Pancreas and Spleen Surgery, The People’s Hospital of Guangxi Zhuang Autonomous Region, Guangxi Academy of Medical Sciences, Nanning, China

**Keywords:** circulating metabolites, risk, breast cancer, mendelian randomization study, relationships

## Abstract

**Objective:**

This study aimed to investigate potential causal relationships between circulating metabolites and breast cancer risk using Mendelian randomization (MR) analysis.

**Materials and Methods:**

Summary-level genome-wide association study (GWAS) datasets for 249 circulating metabolites were obtained from the UK Biobank. GWAS datasets for estrogen receptor-positive (ER+) and estrogen receptor-negative (ER-) breast cancer were acquired from previous studies based on the Combined Oncoarray. Instrumental variables (IVs) were selected from single nucleotide polymorphisms (SNPs) associated with circulating metabolites, and MR analyses were conducted using the inverse-variance weighted (IVW) method as the primary analysis, with additional sensitivity analyses using other MR methods. Odds ratios (OR) and 95% confidence interval (CI) were used to estimate the association of circulating metabolites with breast cancer risk.

**Results:**

The IVW analysis revealed significant causal relationships between 79 circulating metabolites and ER + breast cancer risk, and 10 metabolites were significantly associated with ER-breast cancer risk. Notably, acetate (OR = 1.12, *P* = 0.03), HDL cholesterol (OR = 1.09, *P* < 0.001), ration of omega-6 fatty acids to total fatty acids ratio (OR = 1.09, *P* = 0.01), and phospholipids in large LDL (OR = 1.09, *P* < 0.001) were linked to an increased risk of ER + breast cancer, while linoleic acid (OR = 0.91, *P* < 0.001) monounsaturated fatty acids (OR = 0.91, *P* < 0.001), and total lipids in LDL (OR = 0.91, *P* < 0.001) were associated with a decreased risk. In ER-breast cancer, glycine, citrate, HDL cholesterol, cholesteryl esters in HDL, cholesterol to total lipids ratio in very large HDL, and cholesterol in large LDL were associated with an increased risk, while the free cholesterol to total lipids in very large HDL was linked to a decreased risk.

**Conclusion:**

This MR approach underscores aberrant lipid metabolism as a key process in breast tumorigenesis, and may inform future prevention and treatment strategies. To further elucidate the underlying mechanisms and explore the potential clinical implications, additional research is warranted to validate the observed associations in this study.

## Introduction

Breast cancer is one of the most prevalent malignancies and is the leading cause of cancer-related mortality among women worldwide (Sung et al., 2021). Despite remarkable advances have been made in breast cancer treatment, primary prevention remains an important strategy to curb the growing burden of this disease. Identification of modifiable risk factors is crucial for developing preventive interventions (Sun et al., 2017).

Emerging evidence suggests that circulating metabolites play important roles in breast cancer development (Asiago et al., 2010; Huang et al., 2016; Subramani et al., 2022). Metabolites represent key molecular readouts of cellular activities and can reflect the pathophysiological status, characterizing metabolites associated with disease risk may shed new light on the etiology of breast cancer (Subramani et al., 2022). Acetate, a short-chain fatty acid produced mainly by gut microbiota, has been implicated in cancer cell proliferation and metastasis (Mashimo et al., 2014; Schug et al., 2016). Studies have shown that acetate can be utilized by cancer cells to fuel lipid synthesis and support rapid tumor growth. HDL cholesterol, traditionally considered protective against cardiovascular diseases, has shown mixed associations with breast cancer risk (Davidson et al., 2021). While some studies suggest that HDL cholesterol may be protective, others indicate a potential role in promoting cancer progression. Linoleic acid, a polyunsaturated omega-6 fatty acid, has also exhibited dual roles, with some evidence pointing to its anti-inflammatory properties and others to its potential to promote tumor growth (Wolk et al., 1998; Zanoaga et al., 2018).

However, current studies are predominately based on case-control designs, which are inadequate to infer causality due to susceptibility to confounding and reverse causation (Burgess et al., 2017). Mendelian randomization (MR) analysis utilizes genetic variants as instrumental variables to infer causality between modifiable exposures and disease outcomes in epidemiological studies (Davey Smith and Hemani, 2014). By leveraging the random assortment of alleles during conception, MR minimizes biases from confounding factors and establishes temporality (Zheng et al., 2017). Several MR studies have provided causal insight into breast cancer risk factors, but none have systematically examined the effects of circulating metabolites (Chen et al., 2022; Escala-Garcia et al., 2020). This study hypothesizes that specific circulating metabolites may either increase or decrease the risk of breast cancer subtypes, with a focus on understanding the differential effects on ER+ and ER-breast cancers. Therefore, we performed comprehensive MR analyses to evaluate putative causal effects of a panel of 249 circulating metabolites on breast cancer risk.

## Materials and methods

### Study design

To explore the effects of plasma metabolites on breast cancer, we respectively conducted MR analyses with genetic instrumentals generated from studies on metabolomics quantitative trait loci of circulating metabolites. We obtained summary-level GWAS datasets from the UK Biobank, which includes 249 circulating metabolites divided into nine crucial groups. These metabolites were chosen based on their availability in the dataset and the potential relevance to cancer, as suggested by prior evidence. We discovered the GWAS datasets of breast cancer at https://gwas.mrcieu.ac.uk/datasets. These studies have informed consent and the local ethical committee’s clearance.

### Data sources

#### Metabolic profile for analyses

Summary-level datasets on 249 circulating metabolites (acetate, acetone, alanine, albumin, apolipoprotein A1/B, cholines, citrate, docosahexaenoic acid, creatinine, glutamine, glycine, HDL cholesterol, LDL cholesterol, cholesterol, histidine, isoleucine, linoleic acid, lactate, omega-3 fatty acids, omega-6 fatty acids, There were 115,078 randomly chosen participants in this study. High-throughput nuclear magnetic resonance (NMR) evaluated metabolic indicators in non-fasting baseline EDTA plasma samples (https://biobank.ndph.ox.ac.uk/ukb/label.cgi?id=220). The indicators contain 168 metabolites (unit, mmol/L) and 81 metabolite proportions, spanning numerous metabolism pathways, including lipoproteins, fatty acids, amino acids, and ketone bodies.

#### IV selection

SNPs associated with the 249 circulating metabolites were selected using a genome-wide significance threshold (*P* < 5*10^−8^). We identified and excluded SNPs that were in linkage disequilibrium (LD) (*R* ^2^ > 0.001 or within 10,000 kb of the 1,000 Genomes European-ancestry Reference Panel). As previously mentioned, we computed F-statistics to look for instruments that were not up to grade, to ensure concerns about weak instruments were minimal.

#### Breast cancer

An earlier GWAS using the Combined Oncoarray, iCOGS yielded summary statistics for breast cancer (Table 1). The ER + breast cancer dataset (https://gwas.mrcieu.ac.uk/datasets/ieu-a-1127/), which included 175,475 people (69,501 ER + breast cancer cases and 105,974 controls) of European ancestry, looked into the connection between up to 10,680,257 genotyped SNPs. The dataset (https://gwas.mrcieu.ac.uk/datasets/ieu-a-1128/), which included 127,442 people (21,468 ER + breast cancer cases and 105,974 controls) of European ancestry, looked at the link between up to 10,680,257 genotyped SNPs and ER-breast cancer. The data above sets are equivalent because they originate from the same database.

**TABLE 1:** Descriptions for data sources and assessment of the instrumental variables strength.

Traits	Sample size	Cases/controls	Race	Year	Data source	Web source
249 Circulating metabolites	1,15,078	NA	Europeans	2020	UK Biobank	https://www.ukbiobank.ac.uk/
ER- Breast cancer	1,27,442	21,468/1,05,974	Europeans	2017	Combined Oncoarray; iCOGS; GWAS meta analysis	https://gwas.mrcieu.ac.uk/datasets/ieu-a-1128/
ER + Breast cancer	1,75,475	69,501/1,05,974	Europeans	2017	Combined Oncoarray; iCOGS; GWAS meta analysis	https://gwas.mrcieu.ac.uk/datasets/ieu-a-1127/

#### Mendelian randomization

The primary technique for estimating causality was the inverse-variance weighted (IVW). We assessed the IVW analyses’ heterogeneities by Cochran’s Q values. Further sensitivity studies included MR-Egger, simple mode, weighted mode, simple median, and weighted median. Due to its ability to identify and account for any horizontal pleiotropy, the MR-Egger technique can provide accurate causal estimations even when pleiotropy is present (*P* for intercept 0.05). The weighted median method supports causal predictions when up to 40% of the weight in the MR analysis originates from inaccurate instrument data. We completed all statistical studies by R with the “TwoSampleMR” package. The version is 3.4.2 (R Foundation for Statistical Computing, Vienna, Austria). A two-tailed *p*-value of <0.05 indicates significant statistics.

## Results

### Instrumental variables for circulating metabolites on breast cancer

**FIGURE 1:**
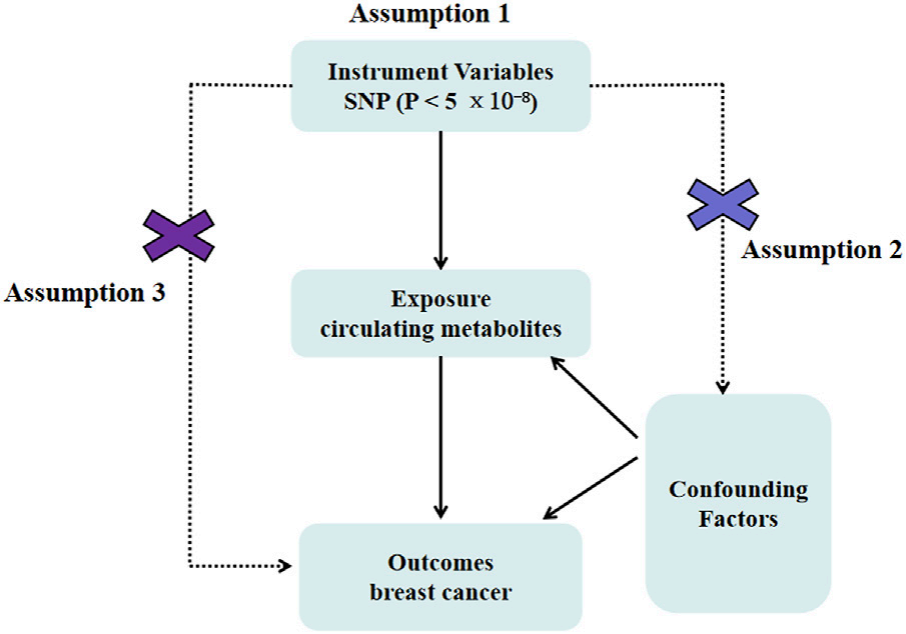
Mendelian randomization model of circulating metabolites and risk of breast cancer. It presents the overall design and abstract of this study’s result.

More than 10,000 SNPs were employed as IVs for 249 circulating metabolites and breast cancer, respectively, based on the selection criteria of IVs. In this investigation, we carried out a two-sample MR analysis to assess the causal relationship between circulating metabolites and breast cancer (Figure 1). Table 1 displays specific information regarding the data sources.

### The causal effect of circulating metabolites on ER + breast cancer

**FIGURE 2:**
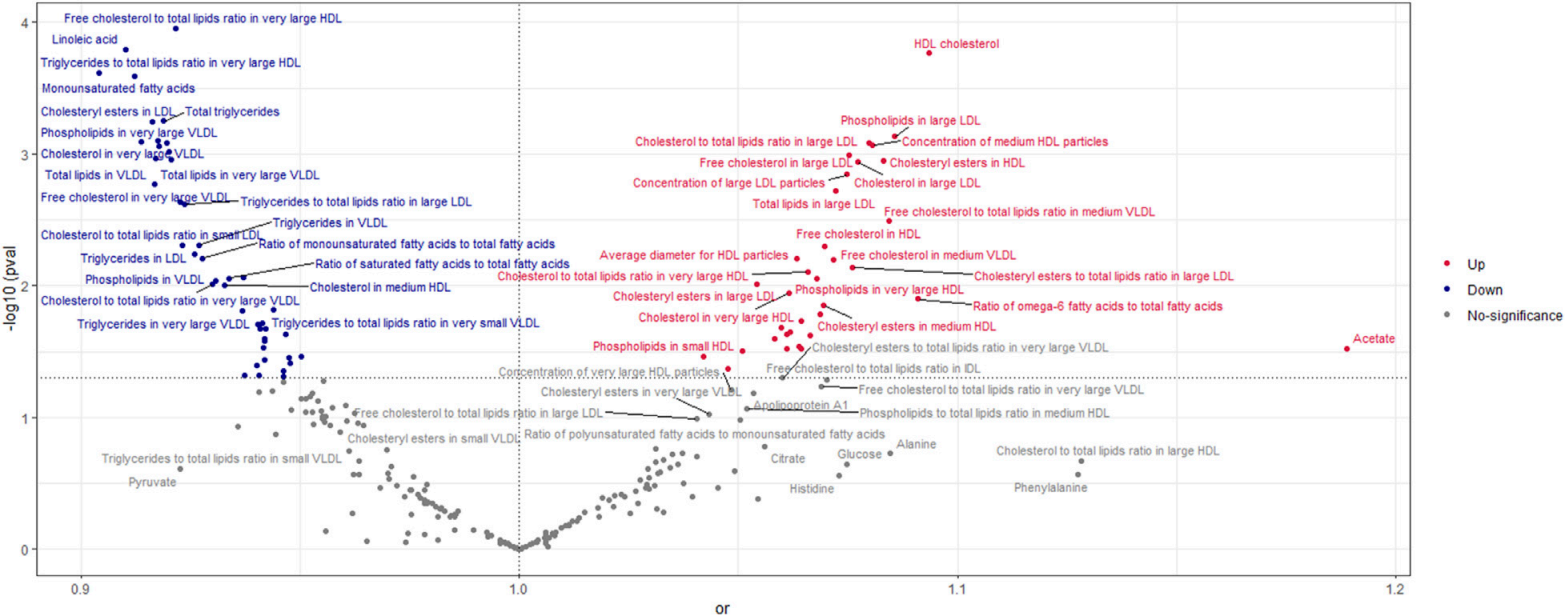
Volcano Plot visualizing the result of IVW in positive breast cancer. The red dots present circulating metabolites performing harmful effects on positive breast cancer. The blue dots are circulating metabolites, significantly reducing the risk of positive breast cancer. The grey dots indicate an insignificant causal impact on positive breast cancer.

According to the findings of the IVW (multiplicative random effects) technique (Supplementary Tables S1, S2), 79 circulating metabolites had a significant causal connection with ER + breast cancer. As shown in Figure 2, the volcanic map provides the preliminary visualization of MR results. The increased risk of ER + breast cancer was linked to acetate, HDL cholesterol, the ratio of omega-6 to total fatty acids, phospholipids in large LDL, cholesteryl esters in HDL, cholesterol in large LDL, free cholesterol in large LDL, and total lipids in large LDL. Linoleic acid, total lipids in VLDL and LDL, and monounsaturated fatty acids were associated with a lower chance of developing ER + breast cancer.

**FIGURE 3:**
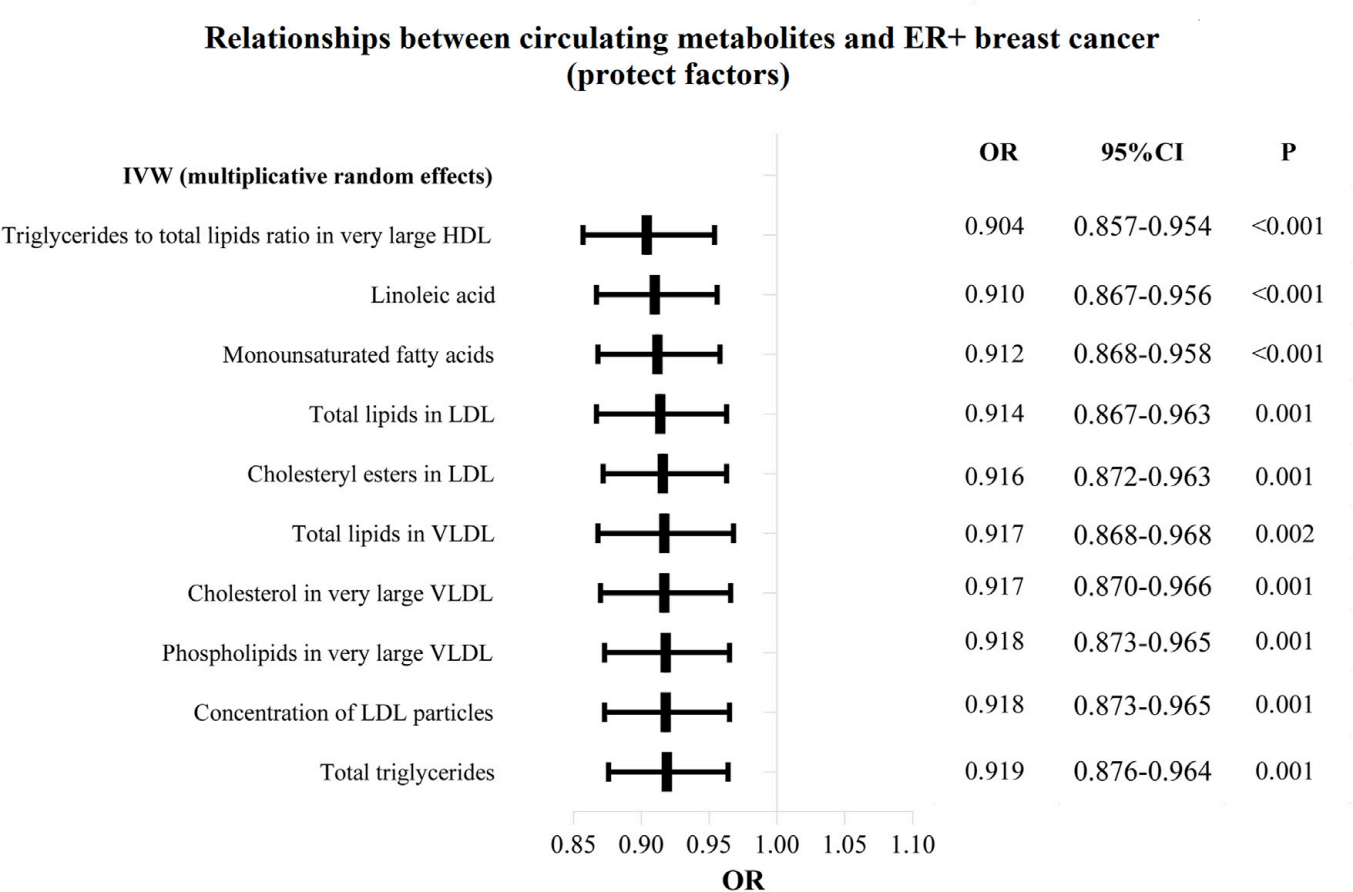
The circulating metabolites with a protective effect on positive breast cancer in IVW (random effects). The black dots and bars indicated the causal estimate and 95% CI of each circulating metabolite on positive breast cancer by random-effect inverse variance weighted method.

**FIGURE 4:**
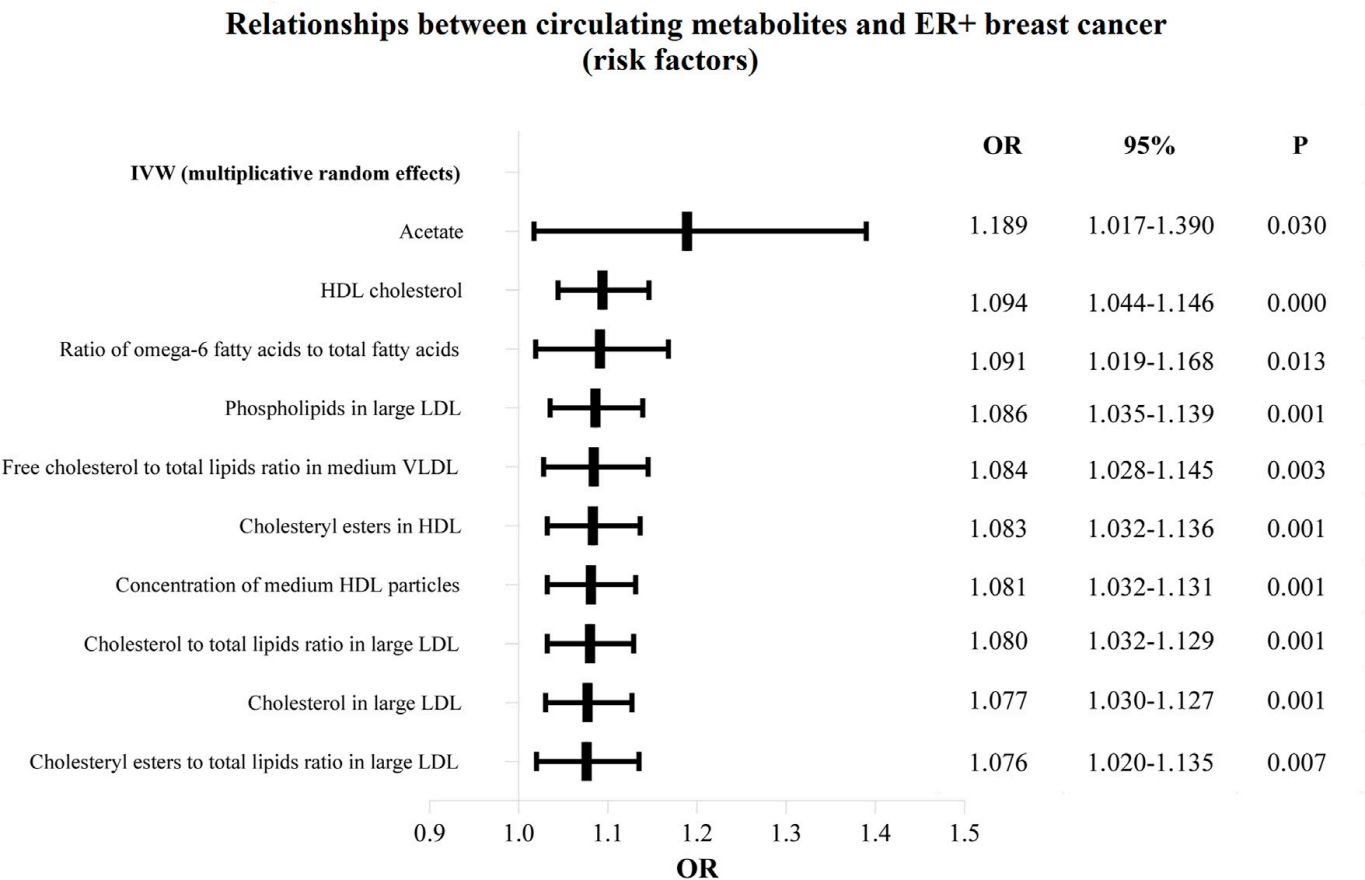
The circulating metabolites with a harmful impact on positive breast cancer in IVW (random effects). The black dots and bars indicated the causal estimate and 95% CI of each circulating metabolite on positive breast cancer by random-effect inverse variance weighted method.

The OR values of circulating metabolites on ER + breast cancer were sequenced and displayed by forest plot (we chose the top 10 findings as the significant results), as shown in Figures 3, 4. The aim is to investigate further the impact of circulating metabolites on ER + breast cancer. We discovered that acetate (OR = 1.120, 95%CI = 1.017–1.390, *p* = 0.030), HDL cholesterol (OR = 1.094, 95%CI = 1.044–1.146, *p* = 0.001), the ratio of omega-6 to total fatty acids (OR = 1.091, 95%CI = 1.019–1.168, *p* = 0.013), phospholipids in large LDL (OR = 1.086, 95%CI = 1.035–1.139, *p* = 0.001). Supplementary Table S3 displays the specific causative relationships between each genetic variation of each circulating metabolite and ER + breast cancer as determined by other MR analysis techniques.

### The causal effect of circulating metabolites on ER-breast cancer

**FIGURE 5:**
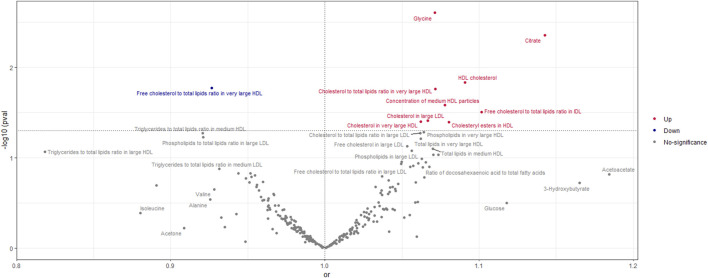
Volcano Plot visualizing the result of IVW in negative breast cancer. The red dots present circulating metabolites increasing the risk of negative breast cancer. The blue dots are circulating metabolites with protective effects on negative breast cancer. The grey dots indicate circulating metabolite without a causal impact on negative breast cancer.

According to the findings of the IVW (multiplicative random effects) technique, which are displayed in Supplementary Tables S4, S5 circulating metabolites, we had a significant causal connection with ER-breast cancer. As shown in Figure 5, the volcanic map provides the early visualization of MR results. The increased risk of ER-breast cancer was linked to glycine, citrate, HDL cholesterol, cholesteryl esters in HDL, cholesterol to total lipids ratio in extensive HDL, and cholesterol in large LDL. In extensive HDL, the proportion of free cholesterol to total lipids was linked to a lower chance of developing ER breast cancer.

**FIGURE 6:**
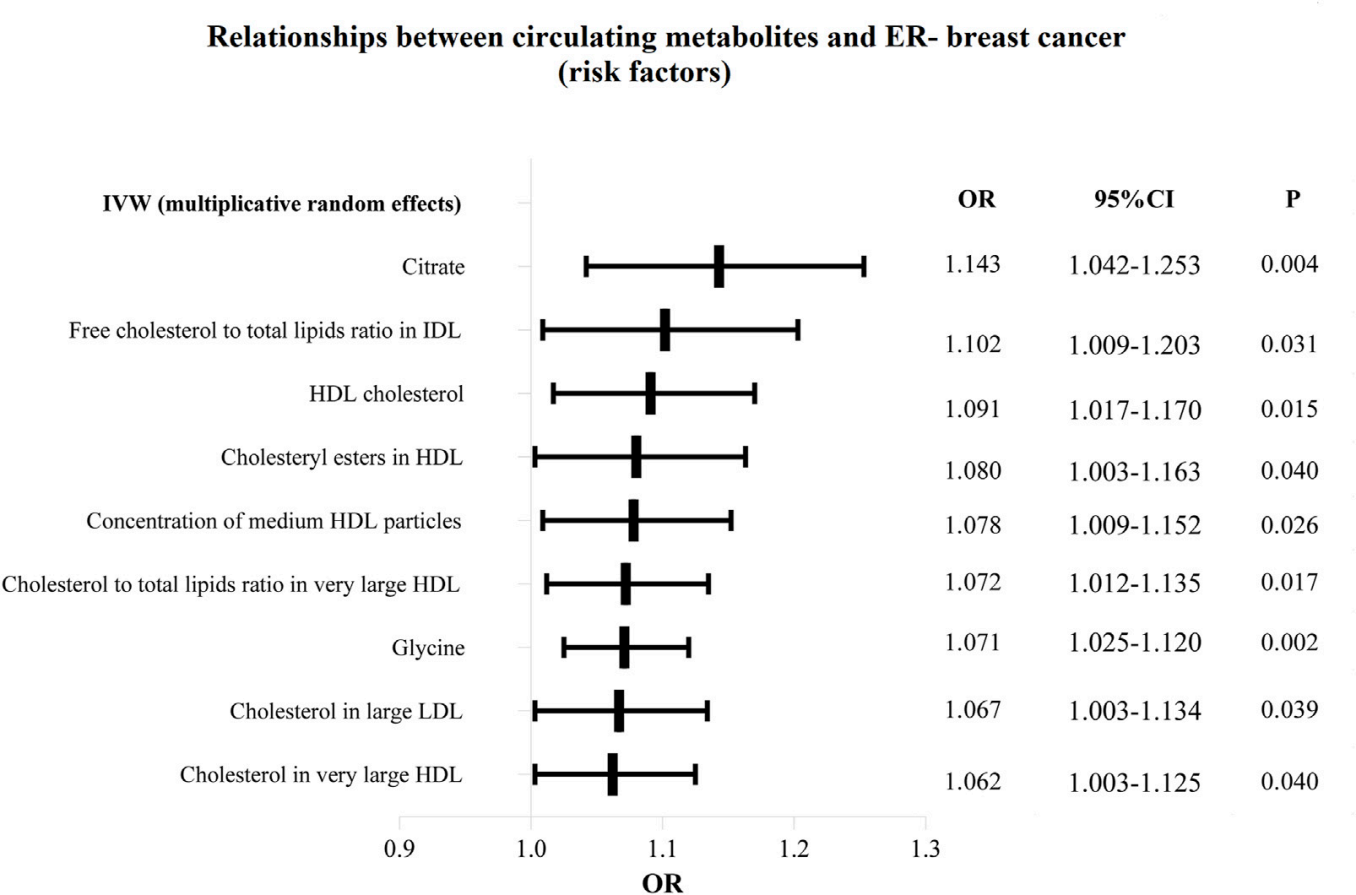
The circulating metabolites with a harmful impact on negative breast cancer in IVW (random effects). The black dots and bars indicated the causal estimate and 95% CI of each circulating metabolite on negative breast cancer by random-effect inverse variance weighted method.

**FIGURE 7:**
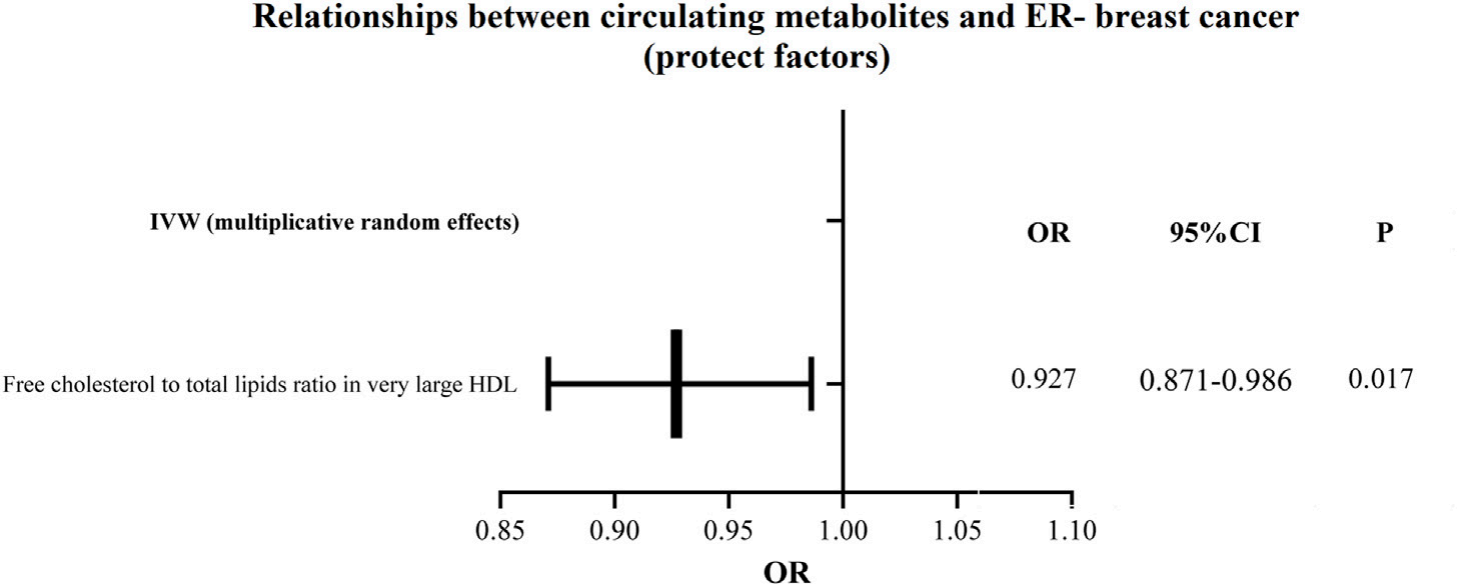
The circulating metabolites with a protective influence on negative breast cancer in IVW (random effects). The black dots and bars indicated the causal estimate and 95% CI of each circulating metabolite on negative breast cancer by random-effect inverse variance weighted method.

Additionally, as shown in Figures 6, 7, we eliminated circulating metabolites with a substantial increase or decrease in risk for ER-breast cancer (*p* = 0.05), sorted them according to OR values, and then displayed them as a forest plot. Citrate (OR = 1.143, 95%CI = 1.042–1.253, *p* = 0.004), Free cholesterol to total lipids ratio in IDL (OR = 1.102, 95%CI = 1.009–1.203, *p* = 0.031), HDL cholesterol (OR = 1.091, 95%CI = 1.017–1.170, *p* = 0.015), Cholesteryl esters in HDL (OR = 1.080, 95%CI = 1.003–1.163, *p* = 0.040). Supplementary Table S6 displays the specific causative relationships between each genetic variation of each circulating metabolite and ER-breast cancer as determined by different MR analysis techniques.

### Secondary results associated with selected instruments and sensitivity analyses

To test the IVW method’s findings, we also performed MR-Egger, simple mode, weighted mode, simple median, weighted median, and penalized weighted median analyses. Due to variations in statistical methods, most of these 249 metabolites in circulation did not provide reverse results; the results are shown in Supplementary Table S3. As can be seen in Supplementary Table S6, we also discovered no appreciable methodological variations leading to unstable results in the ER-breast cancer dataset.

### The heterogeneity and horizontal pleiotropy test results of circulating metabolites in breast cancer

More than half of the causative relationship between circulating metabolites and ER + breast cancer appeared heterogeneous (*P* for Cochran’s Q of IVW 0.05) which was shown in Supplementary Table S7. Supplementary Table S8 shows heterogeneity in the causative connection between circulating metabolites and ER-breast cancer (*P* for Cochran’s Q of IVW 0.05). In light of this, we select IVW (multiplicative random effects) as our primary analytic approach and create a forest map based on the findings.

The MR-Egger Intercept test demonstrated that horizontal pleiotropy had no discernible impact on our findings. As shown in Supplementary Table S9, most of the MR-Egger intercept test’s *p* values for ER + breast cancer circulating metabolites were more than 0.05.

The MR-Egger Intercept test demonstrated that horizontal pleiotropy did not impact our findings. Except for the ratio of omega-6 fatty acids to total fatty acids (*p* = 0.017), the percentage of free cholesterol to total lipids in chylomicrons and huge VLDL (*p* = 0.017), and the ratio of tyrosine (*p* = 0.047), none of the circulating metabolites on ER-breast cancer had a significant impact on our final finding, as shown in Supplementary Table S10.

## Discussion

In this study, we performed systematic MR analyses to investigate causal associations of 249 circulating metabolites with breast cancer risk, focusing on ER+ and ER-subtypes. Our findings provide valuable evidence suggesting that alterations in systemic metabolite levels may play an integral role in breast carcinogenesis and progression.

The positive association between acetate and ER + breast cancer risk is consistent with previous findings that acetate plays a significant role in cancer cell metabolism (Mashimo et al., 2014; Schug et al., 2016), where it serves as an important bioenergetic substrate fueling lipid synthesis, thereby promoting tumorigenesis and metastasis. Acetate, primarily derived from gut microbial fermentation, has emerged as a key metabolite involved in cancer cell proliferation and metastasis (Rodríguez-Enríquez et al., 2021). Acetate can be taken up by cancer cells and utilized for fatty acid synthesis to meet the demands of rapid growth (Comerford et al., 2014). Conceivably, elevated systemic acetate levels may stimulate proliferative signaling pathways and confer a more aggressive phenotype in ER + breast cancer (You et al., 2023). However, additional in-depth mechanistic studies are required to dissect the mutagenic mechanisms.

Additionally, dysregulated HDL cholesterol and LDL lipid levels were found to be intricately linked with both ER+ and ER-breast cancer risk. Although dyslipidemia is linked to postmenopausal breast cancer risk, previous epidemiological data on HDL cholesterol have been inconsistent (Ni et al., 2015; Johnson et al., 2020), the clinical relevance of HDL cholesterol may extend beyond its absolute levels, as the composition and function of HDL particles have been found to play crucial roles (Davidson et al., 2021; Pirillo et al., 2013). Similarly, while phospholipids in large LDL were associated with increased ER + breast cancer risk, total lipids in LDL showed protective effects in our analysis. These contrasting relationships of HDL cholesterol, LDL lipids, and their subfractions with breast cancer risk align with emerging evidence that HDL and LDL particles have context-dependent, pleiotropic effects depending on composition and function (Cedó et al., 2019). Further exploration of specific HDL and LDL particle subtypes and their functions could provide deeper insights into breast carcinogenesis.

Our findings further suggested that linoleic acid and monounsaturated fatty acids may exert protective effects against ER + breast cancer, consistent with their documented anti-inflammatory properties (Wang and Dubois, 2010). However, the impact of linoleic acid and monounsaturated fatty acids on breast cancer development and progression is an area still under exploration (MacLennan and Ma, 2010). Previous research has demonstrated that polyunsaturated fatty acids, including linoleic acid, have been shown to suppress cancer cell growth by downregulating oncogenic signaling pathways (Zanoaga et al., 2018). Conversely, it is found that a higher intake of ω-6 polyunsaturated fatty acids has been associated with an increased risk of various cancers, including breast cancer (Shapira, 2017). Additionally, one study reported a significant positive association between polyunsaturated fat and invasive breast cancer risk, while monounsaturated fat exhibited a significant inverse relationship (Wolk et al., 1998). Furthermore, oleic acid esters, belonging to the omega-9 monounsaturated fatty acid group, were found to stimulate breast cancer cell invasion into the lung (Blücher and Stadler, 2017). The divergent findings on linoleic acid and monounsaturated fatty acids may arise from differences in study populations, methodologies, or the specific contexts in which linoleic acid or monounsaturated fatty acids operate, this underscores the need for further research to elucidate the mechanistic pathways through which linoleic acid and other polyunsaturated fatty acids modulate breast cancer risk, particularly focusing on their interactions with inflammatory processes and lipid metabolism (Fu et al., 2020).

For ER-breast cancer, we found positive associations with glycine, citrate, HDL cholesterol, cholesteryl esters in HDL, and cholesterol in large LDL. Glycine metabolism has been implicated in cancer pathogenesis (Amelio et al., 2014), and citrate is a key metabolite often altered in cancer (Icard et al., 2021).

Collectively, our findings underscore the importance of lipid metabolism, particularly the roles of acetate, HDL, LDL, and fatty acids, as an integral process underlying breast tumorigenesis across ER subtypes. Breast cancer heterogeneity emphasizes the need to elucidate the intricate mechanisms connecting lipid pathways with breast oncogenesis to refine risk prediction and develop targeted prevention and therapy strategies (Fu et al., 2020; Zipinotti dos Santos et al., 2023; Vasseur and Guillaumond, 2022).

Several limitations should be acknowledged when interpreting our findings. First, the MR assumptions need to be validated. Although sensitivity analyses yielded consistent results, pleiotropy cannot be definitively excluded (Davey Smith and Hemani, 2014). Second, our MR approach estimates the cumulative effects of lifelong metabolite exposures, which may not fully capture dynamic changes in metabolite levels (Kettunen et al., 2016). Additional epidemiological studies with repeated metabolite measurements are warranted. Finally, as our study was restricted to individuals of European descent, the generalizability to other ethnicities requires further evaluation.

## Conclusion

In summary, our multi-omics MR approach highlights aberrant lipid metabolism as a pivotal process in breast tumorigenesis. By integrating metabolomics into MR frameworks, we elucidated key metabolic pathways implicated in breast oncogenesis and progression. These findings may ultimately refine risk prediction models, identify biomarkers for early detection, and offer potential targets for breast cancer prevention and therapy. In conclusion, our study provides novel evidence supporting causal associations of multiple circulating metabolites, particularly acetate and linoleic acid, with breast cancer risk in a subtype-specific manner. Our findings offer new insights into the metabolic pathways involved in breast cancer development.

## Data Availability

The original contributions presented in the study are included in the article/Supplementary Material, further inquiries can be directed to the corresponding authors.

## Abbreviations

MD, Mendelian randomization; GWAS, genome-wide association study; ER+, estrogen receptor-positive; ER-, estrogen receptor-negative; IVs, Instrumental variables; SNPs, single nucleotide polymorphisms; IVW, inverse-variance weighted; OR, Odds ratios; CI, confidence interval; NMR, nuclear magnetic resonance.
